# Author Correction: R-loop-dependent promoter-proximal termination ensures genome stability

**DOI:** 10.1038/s41586-025-08606-x

**Published:** 2025-01-20

**Authors:** Congling Xu, Chengyu Li, Jiwei Chen, Yan Xiong, Zhibin Qiao, Pengyu Fan, Conghui Li, Shuangyu Ma, Jin Liu, Aixia Song, Bolin Tao, Tao Xu, Wei Xu, Yayun Chi, Jingyan Xue, Pu Wang, Dan Ye, Hongzhou Gu, Peng Zhang, Qiong Wang, Ruijing Xiao, Jingdong Cheng, Hai Zheng, Xiaoli Yu, Zhen Zhang, Jiong Wu, Kaiwei Liang, Yan-Jun Liu, Huasong Lu, Fei Xavier Chen

**Affiliations:** 1 Fudan University Shanghai Cancer Center, Institutes of Biomedical Sciences, State Key Laboratory of Genetic Engineering, Shanghai Key Laboratory of Medical Epigenetics, Shanghai Key Laboratory of Radiation Oncology, Human Phenome Institute, Fudan University, Shanghai, China; 2 Zhejiang Provincial Key Laboratory for Cancer Molecular Cell Biology, Life Sciences Institute, Zhejiang University, Hangzhou, China; 3 Department of Radiation Oncology, Fudan University Shanghai Cancer Center, Fudan University, Shanghai, China; 4 Department of Thoracic Surgery, Shanghai Pulmonary Hospital, Tongji University School of Medicine, Shanghai, China; 5 Department of Pathophysiology, School of Basic Medical Sciences, Wuhan University, Wuhan, China; 6 Department of Histoembryology, Genetics and Developmental Biology, Shanghai Key Laboratory of Reproductive Medicine, Key Laboratory of Cell Differentiation and Apoptosis of Chinese Ministry of Education, Shanghai Jiao Tong University School of Medicine, Shanghai, China; 7 Inflammation and Immune Mediated Diseases Laboratory of Anhui Province, Anhui Institute of Innovative Drugs, School of Pharmacy, Anhui Medical University, Hefei, China; 8 Department of Orthopedic Oncology, Changzheng Hospital, Second Military Medical University, Shanghai, China; 9 Department of Breast Surgery, Fudan University Shanghai Cancer Center, Shanghai, China; 10 Huashan Hospital, Fudan University, Shanghai Key Laboratory of Medical Epigenetics, Molecular and Cell Biology Lab, Institutes of Biomedical Sciences, Shanghai Medical College of Fudan University, Shanghai, China

**Keywords:** Chromatin, Transcription

Correction to: *Nature* https://www.nature.com/articles/s41586-023-06515-5 Published online 9 August 2023

In the version of the article initially published, in Fig. 1e, the western blot probed with the PP2A-C antibody was inadvertently duplicated from the blot probed with the INTS4 antibody. This error occurred due to the mistaken use of an incorrect uncropped image during manuscript preparation. We have now traced the correct uncropped image and updated Fig. 1e, along with Supplementary Fig. 1, which contains the source data for the unprocessed western blots. Figure 2j should display heatmaps of Pol II ChIP-seq for CTR and DKO cells, as described in the corresponding figure legend. However, during the final round of revision, this panel was inadvertently replaced with one showing sgCtr and INTS2-KO cells. We have now updated Fig. 2j to include the correct heatmaps. The treatment conditions for the last (6th) column in Fig. 3g should match those in the last (6th) column of Fig. 3f. The final version we submitted was correct. However, the labelling for Fig. 3g was incorrect in the published version. We have now updated Fig. 3g with the correct labelling as well. The original and corrected Figs. 1 and 2 and Supplementary Fig. 1 can be seen in the Supplementary information accompanying this amendment. These corrections have been made to the HTML and PDF versions of the article.

## Supplementary information

Original and corrected Figs. 1 and 2 and Supplementary Fig. 1

