# Supplementary material for: R-loop-dependent promoter-proximal termination ensures genome stability
Source: Nature. 2023 Aug 9;621(7979):610–9. doi: 10.1038/s41586-023-06515-5 (PMC10511320; doi:10.1038/s41586-023-06515-5)
Supplement: Supplementary file 1 — Supplementary Figs. 1–6, Supplementary Note 1 and legends for Supplementary Tables 1–3. [file 41586_2023_6515_MOESM1_ESM.pdf]

nature portfolio

https://doi.org/10.1038/s41586-023-06515-5

Supplementary information

R-loop-dependent promoter-proximal termination ensures genome stability

In the format provided by the authors and unedited

Supplementary Figure 1

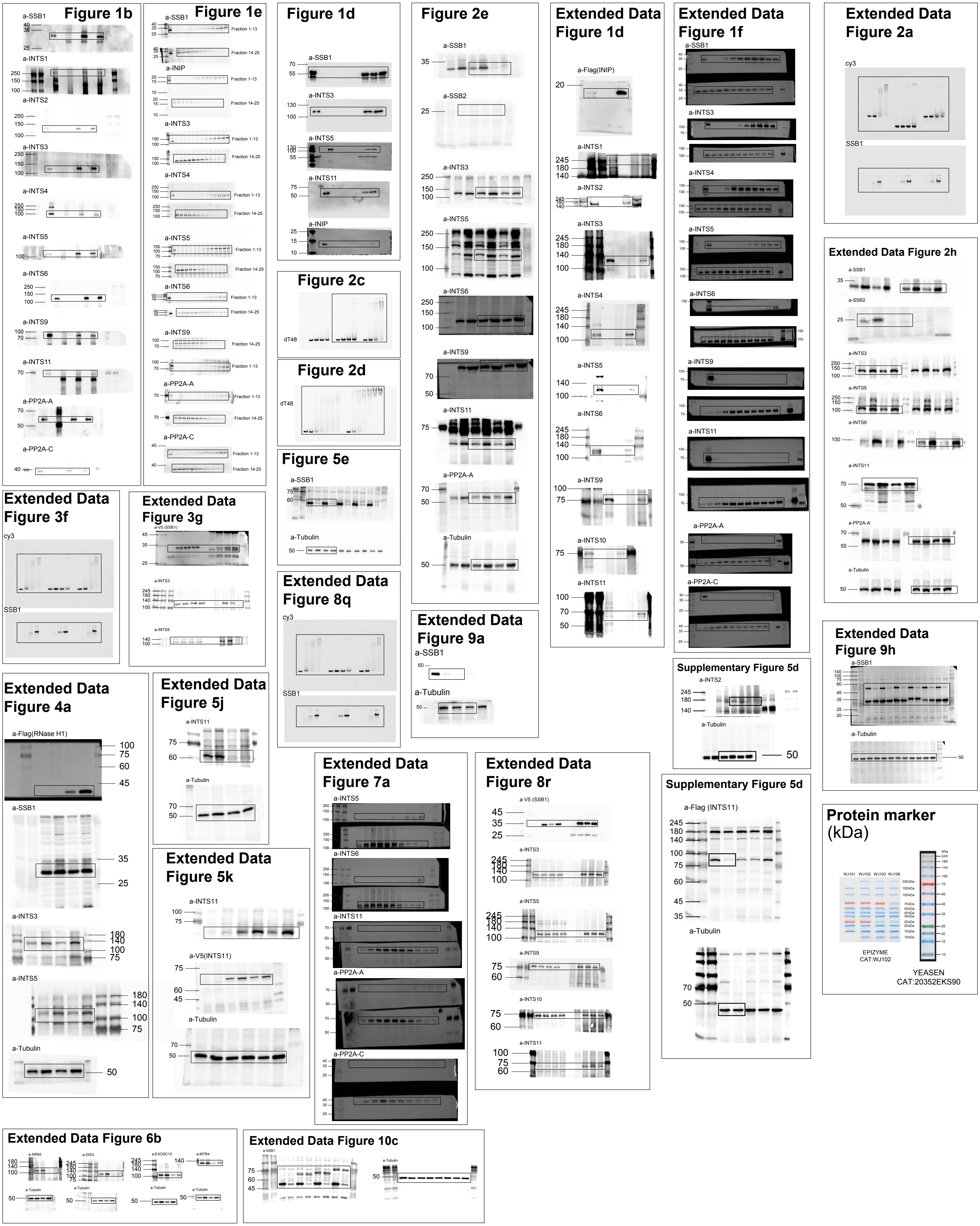

Supplementary Fig. 1. Source Data of unprocessed western blots.

Supplementary Figure 2

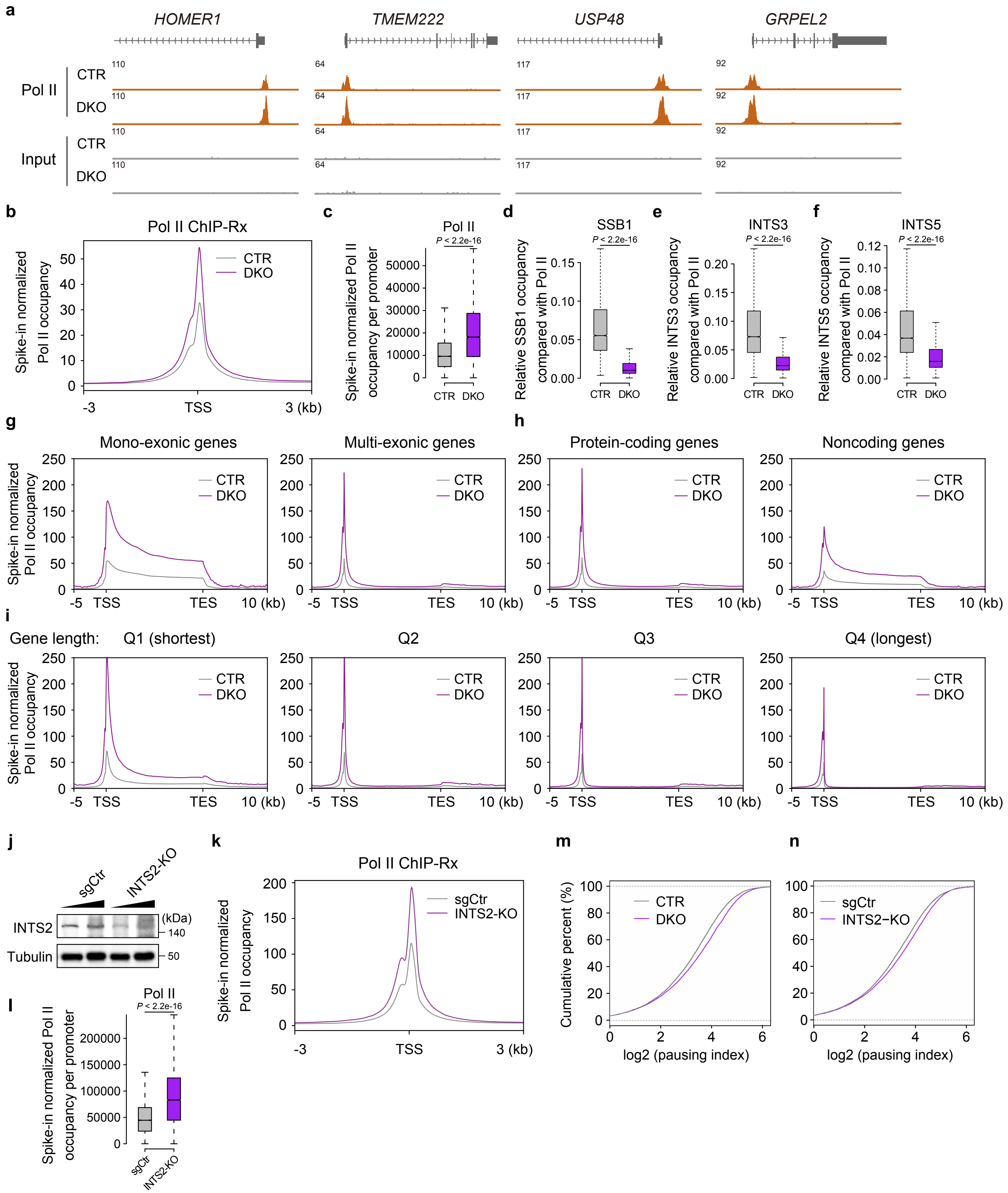

Supplementary Fig. 2. SOSS-INTAC regulates Pol II pausing for different classes of genes.

Supplementary Fig. 2. SOSS-INTAC regulates Pol II pausing for different classes of genes.

(a) Representative browser tracks showing the ChIP-Rx signals of Pol II in CTR and DKO cells.

(b) Metaplot of Pol II signals over 6 kb regions centered on TSS of SOSS-INTAC target genes in CTR and DKO cells.

(c) Boxplots showing the comparison of Pol II signals at SOSS-INTAC target promoters between CTR and DKO cells. In boxplots, the centre line is the median, the top and bottom hinges correspond to the first and third quartiles, respectively, whiskers extend to quartiles ± 1.5 × interquartile range. *P* values were calculated using two-sided Wilcoxon tests. $P < 2.2e-16$, n = 10,650 promoters.

(d-f) Boxplots showing the relative occupancies of SSB1 (d), INTS3 (e) and INTS5 (f) compared with Pol II in CTR and DKO cells. In boxplots, the centre line is the median, the top and bottom hinges correspond to the first and third quartiles, respectively, whiskers extend to quartiles ± 1.5 × interquartile range. *P* values were calculated using two-sided Wilcoxon tests. $P < 2.2e-16$, n = 10,650 promoters for all three boxplots.

(g-i) Metagene analysis of Pol II occupancy in CTR and DKO cells for mono-exonic vs. multi-exonic genes (g), protein-coding vs. non-coding genes (h), and quartiles of genes classified by gene length (i).

(j) Western blotting showing the efficiency of INTS2 KO in pooled DLD-1 cells.

(k) Metaplot of Pol II signals over 6 kb regions centered on TSS of SOSS-INTAC target genes in sgCtr and INTS2-KO DLD-1 cells.

(l) Boxplots showing the comparison of Pol II signals at SOSS-INTAC target promoters between sgCtr and INTS2-KO DLD-1 cells. In boxplots, the centre line is the median, the top and bottom hinges correspond to the first and third quartiles, respectively, whiskers extend to quartiles ± 1.5 × interquartile range. *P* values were calculated using two-sided Wilcoxon tests. $P < 2.2e-16$, n = 10,650 promoters.

(m-n) Empirical cumulative distribution function plot of the pausing index (PI) distribution in CTR and DKO (m), or in sgCtr and INTS2-KO DLD-1 cells (n).

Supplementary Figure 3

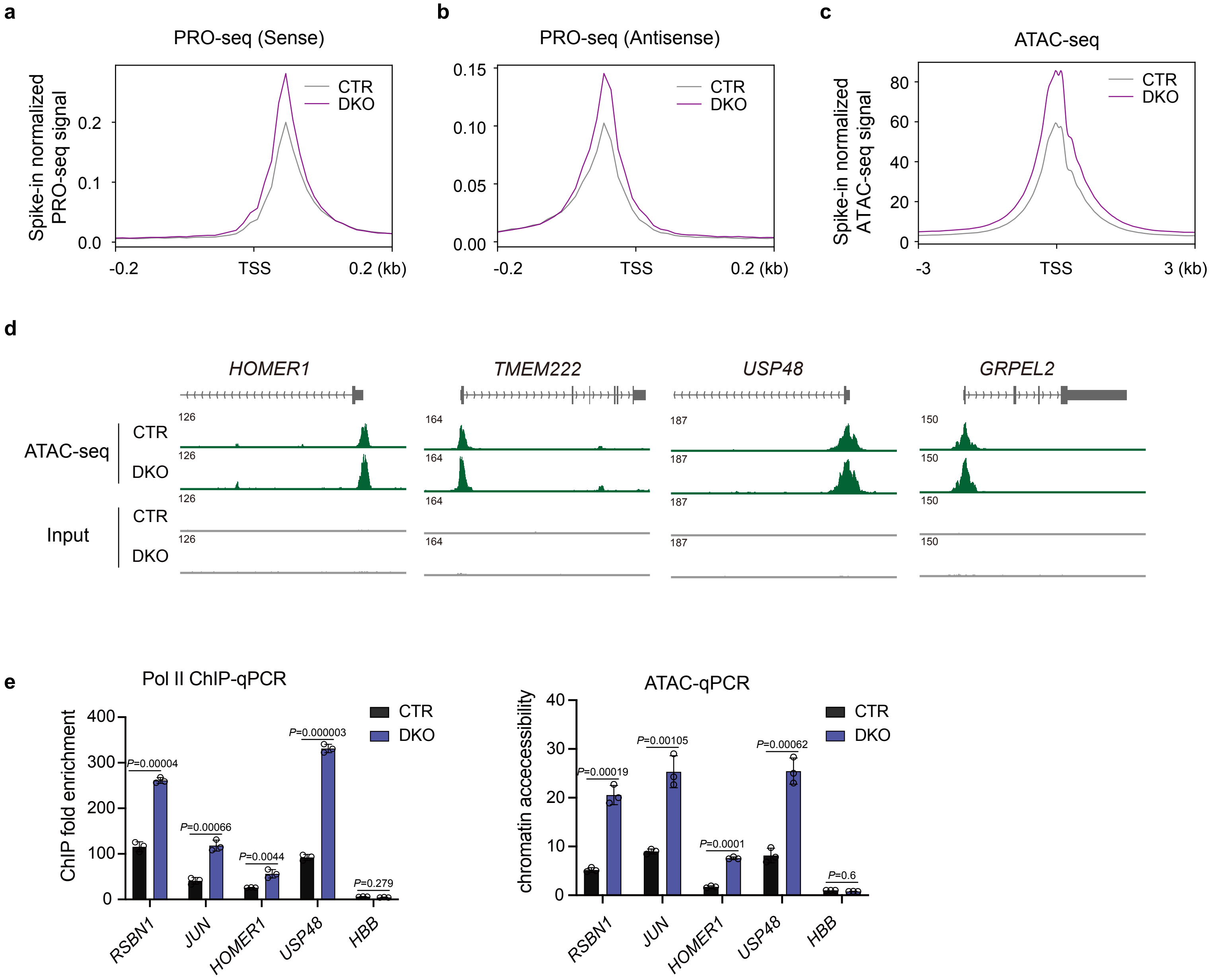

Supplementary Fig. 3. Analysis of PRO-seq, ATAC-seq, and Pol II occupancy in CTR and DKO cells.

(a-b) Metaplots of PRO-seq signals for sense (a) and antisense (b) transcripts over 400 bp regions centered on TSS of SOSS-INTAC target genes in CTR and DKO cells.

(c) Metaplots of ATAC-seq signals over 6 kb regions centered on TSS of SOSS-INTAC target genes in CTR and DKO cells.

(d) Representative browser tracks showing the ATAC-seq signals in CTR and DKO cells.

(e) Pol II ChIP-qPCR (left) and ATAC-qPCR (right) on promoters of example genes in CTR and DKO cells. Values are mean ± SD (n = 3). Statistical analyses were performed using two-tailed unpaired t-test. *P* values are shown at the top of the graphs.

Supplementary Figure 4

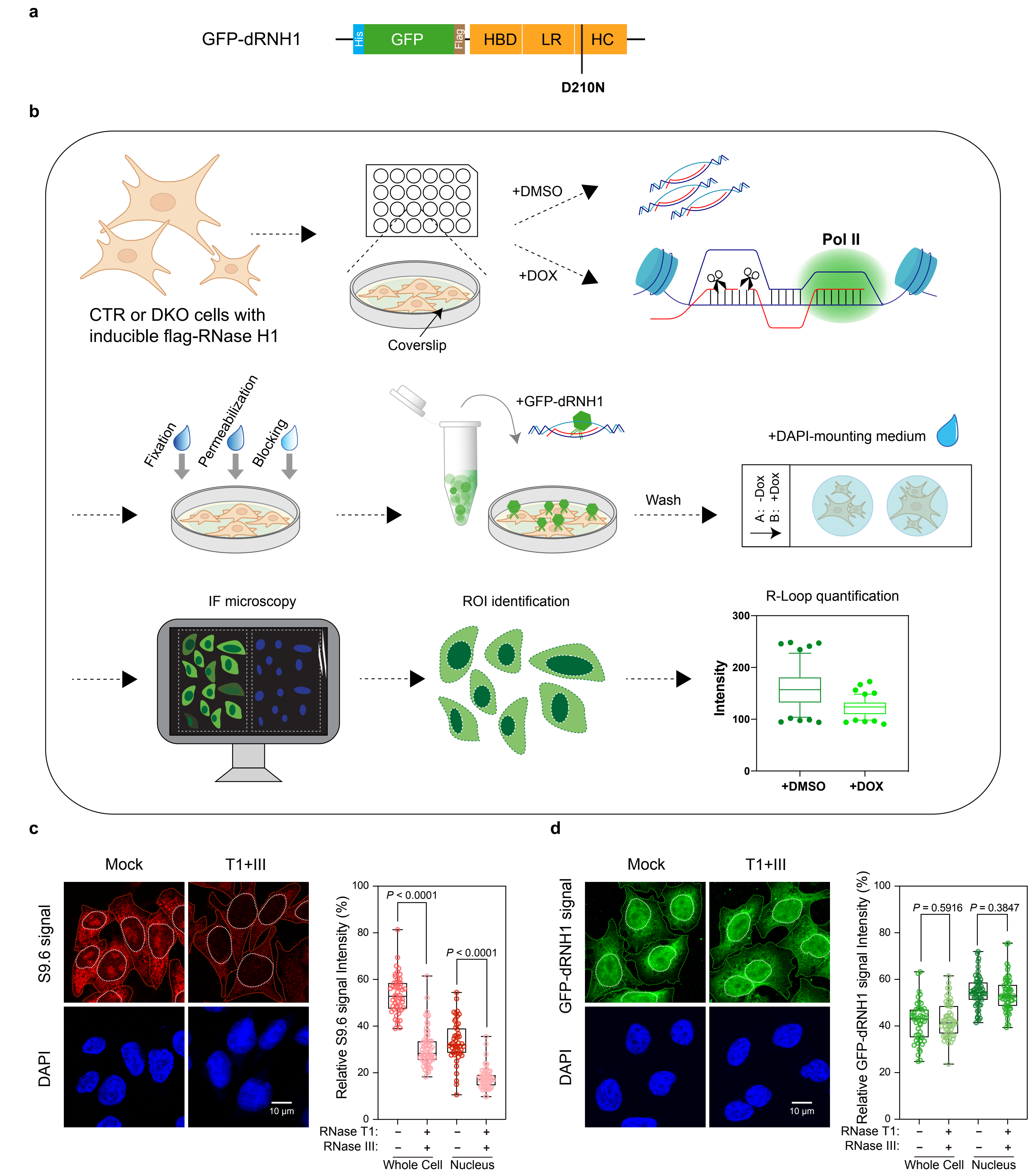

Supplementary Fig. 4. Strategies for R-loop quantification.

(a) Illustration of the GFP-dRNH1 fusion construct used as the sensor for R-loop immunofluorescence.

(b) Schematic presentation of rapid R-loop immunofluorescence workflow.

(c-d) S9.6-based (c) and GFP-dRNH1 (d) R-loop IF pretreated with ssRNA endonuclease RNase T1 and dsRNA endonuclease RNase III in DLD-1 cells.

Supplementary Figure 5

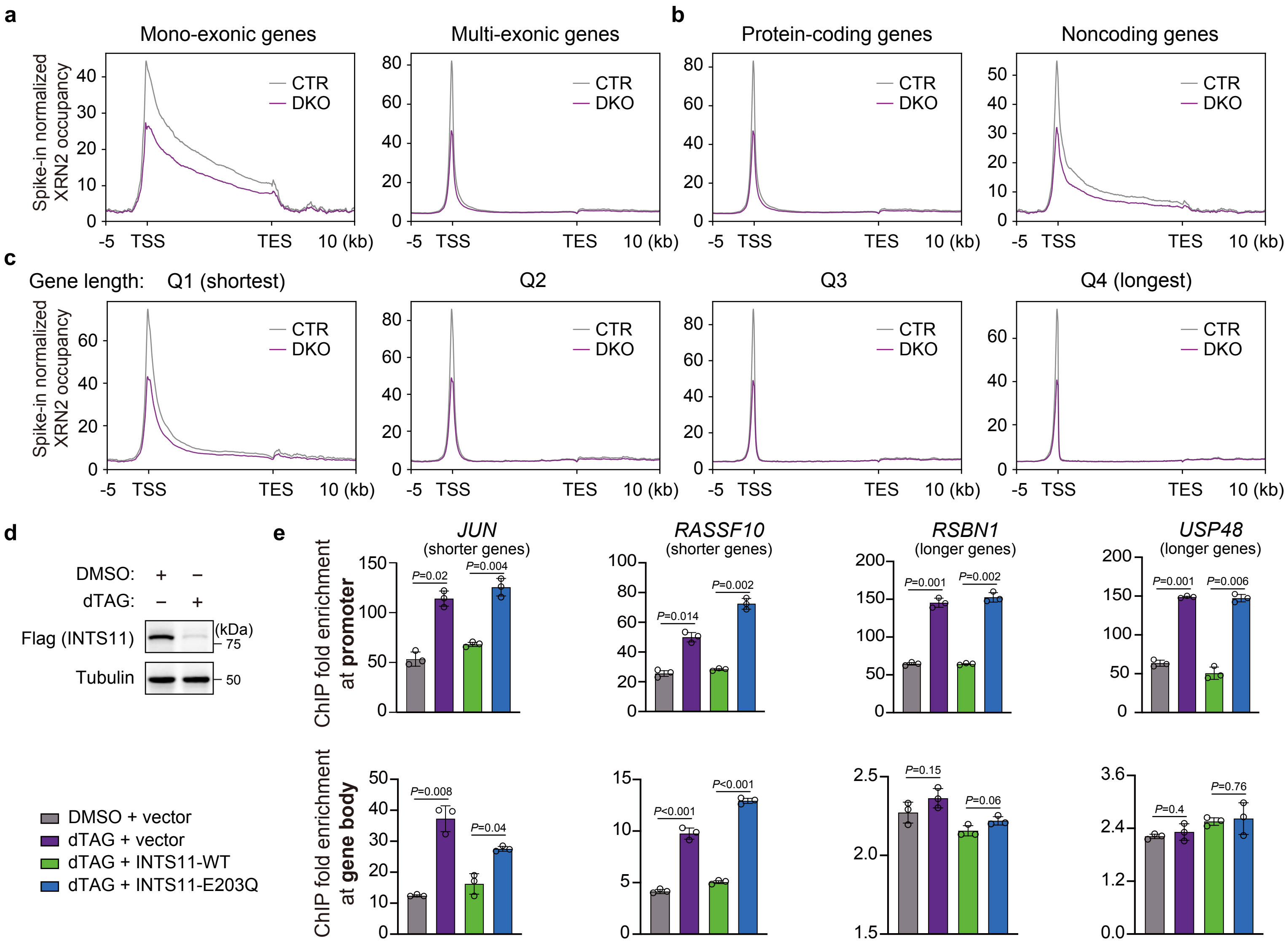

Supplementary Fig. 5. The regulation of XRN2 and Pol II occupancy by SOSS-INTAC for different classes of genes.

(a-c) Metagene analysis of XRN2 occupancy in CTR and DKO cells for mono-exonic vs. multi-exonic genes (a), protein-coding vs. non-coding genes (b), and quartiles of genes classified by gene length (c).

(d) The establishment of INTS11-dTAG DLD-1 cells and verification of degradation efficiency by 12-hour DMSO or dTAG treatment.

(e) Pol II ChIP-qPCR at promoter (top) and gene body (bottom) of example genes (JUN and RASSF10 as shorter genes; RSBN1 and USP48 as longer genes) in DMSO- or dTAG- treated INTS11-dTAG cells with overexpression of empty vector, wild-type or catalytic-dead (E203Q) INTS11. Values are mean ± SD (n = 3). Statistical analysis was performed using two-tailed t-tests. *P* values are shown at the top of the graphs.

Supplementary Figure 6

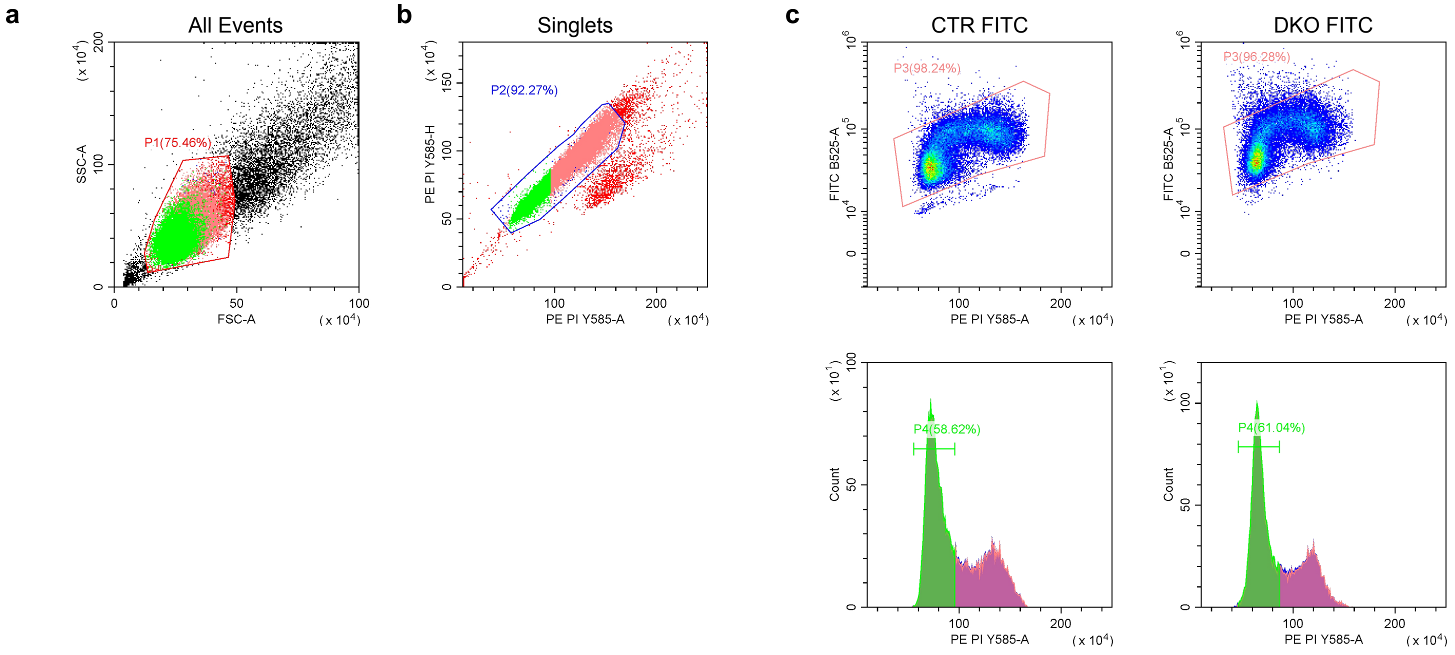

Supplementary Fig. 6. Gating strategy for flow cytometry analysis.

(a) Representative gating for cells in DLD-1 cells.

(b) Representative gating for singlets in DLD-1 cells.

(c) Representation of γH2AX-positive cells in CTR and DKO DLD-1 cells (above). The cell cycle was determined by PI signal (below).

Supplementary Note 1

The calculation formula for the scale factor for ChIP-Rx analysis.

A new scale factor α for each IP experiment was derived as follows:

Let:

- α = The scale factor
- β = The scale factor used for scaling actual mixing ratio
- $N_{IP\_spikein}$ = The spike-in read counts in IP experiment (In millions)
- R_{input} = The actual mixing ratio of IP-corresponding input sample (The relative ratio between spike-in read

counts and experimental read counts, same as that of corresponding IP sample)

- R_{Ref_input} = The actual mixing ratio of reference input sample (Randomly selected, same as that of

corresponding IP sample)

Then, scale factor was calculated as:

$$\alpha = \frac{1}{\beta * N_{IP\_spikein}}$$

Assuming the same mixing ratio among the experiments, we can derive β as:

$$R_{Ref\_input} = \beta * R_{input}$$

or

$$\beta = \frac{R_{Ref\_input}}{R_{input}}$$

Finally, we can derive

$$\alpha = \frac{1}{\frac{R_{Ref\_input}}{R_{input}} * N_{IP\_spikein}}$$

Supplementary tables (1-3), as a combined excel file

- Supplementary table 1. Reagents and resources (tab 1).
- Supplementary table 2. Sequences of oligonucleotides and cDNAs (tab 2).
- Supplementary table 3. Detailed information of high-throughput sequencing experiments (tab 3).
