# Supplementary material for: R-loop-dependent promoter-proximal termination ensures genome stability
Source: Nature. 2023 Aug 9;621(7979):610–9. doi: 10.1038/s41586-023-06515-5 (PMC10511320; doi:10.1038/s41586-023-06515-5)
Supplement: Supplementary file 2 — Reporting Summary [file 41586_2023_6515_MOESM2_ESM.pdf]

nature portfolio

Corresponding author(s): Fei Xavier Chen?Huasong Lu

Last updated by author(s): June 9, 2023

Reporting Summary

Nature Portfolio wishes to improve the reproducibility of the work that we publish. This form provides structure for consistency and transparency in reporting. For further information on Nature Portfolio policies, see our Editorial Policies and the Editorial Policy Checklist.

Statistics

For all statistical analyses, confirm that the following items are present in the figure legend, table legend, main text, or Methods section.

n/a	Confirmed	
☐	☒	The exact sample size (*n*) for each experimental group/condition, given as a discrete number and unit of measurement
☐	☒	A statement on whether measurements were taken from distinct samples or whether the same sample was measured repeatedly
☐	☒	The statistical test(s) used AND whether they are one- or two-sided<br>*Only common tests should be described solely by name; describe more complex techniques in the Methods section.*
☒	☐	A description of all covariates tested
☐	☒	A description of any assumptions or corrections, such as tests of normality and adjustment for multiple comparisons
☐	☒	A full description of the statistical parameters including central tendency (e.g. means) or other basic estimates (e.g. regression coefficient) AND variation (e.g. standard deviation) or associated estimates of uncertainty (e.g. confidence intervals)
☐	☒	For null hypothesis testing, the test statistic (e.g. *F*, *t*, *r*) with confidence intervals, effect sizes, degrees of freedom and *P* value noted<br>*Give P values as exact values whenever suitable.*
☒	☐	For Bayesian analysis, information on the choice of priors and Markov chain Monte Carlo settings
☐	☒	For hierarchical and complex designs, identification of the appropriate level for tests and full reporting of outcomes
☐	☒	Estimates of effect sizes (e.g. Cohen's *d*, Pearson's *r*), indicating how they were calculated

Our web collection on statistics for biologists contains articles on many of the points above.

Software and code

Policy information about availability of computer code

Data collection	Expression of SSB1 and SSB2 across tissues were analyzed by GTEx (https://gtexportal.org/home/). SSB1 mutations in human cancer were analyzed by COSMIC (https://cancer.sanger.ac.uk/cosmic).
Data analysis	Disordered regions were identified using IUPred v2A (http:// iupred.elte.hu/).<br>Amino acid composition was analyzed using Composition Profiler (http://www.cprofiler.org/cgi-bin/profiler.cgi).<br>The net charge per residue was analyzed by CIDER 40 (http://pappulab.wustl. edu/CIDER/analysis/).<br>Images were acquired by confocal microscope (Lecia TCS SP8) and analyzed with software ZEN v2.3 SP1 (ZEISS).<br>Statistical analysis was performed using Prism v8.

For manuscripts utilizing custom algorithms or software that are central to the research but not yet described in published literature, software must be made available to editors and reviewers. We strongly encourage code deposition in a community repository (e.g. GitHub). See the Nature Portfolio guidelines for submitting code & software for further information.

Data

Policy information about availability of data

All manuscripts must include a data availability statement. This statement should provide the following information, where applicable:

- Accession codes, unique identifiers, or web links for publicly available datasets
- A description of any restrictions on data availability
- For clinical datasets or third party data, please ensure that the statement adheres to our policy

The high-throughput sequencing data, including ChIP-Rx, KAS-seq, PRO-seq, ATAC-seq and CUT&Tag, have been deposited in Gene Expression Omnibus database with the accession number GSE223997. The scripts used to analyze the data from this study are freely available at: https://github.com/chenjiwei124128/SSB1_NGS_analysis

Human research participants

Policy information about studies involving human research participants and Sex and Gender in Research.

Reporting on sex and gender	N/A
Population characteristics	N/A
Recruitment	N/A
Ethics oversight	N/A

Note that full information on the approval of the study protocol must also be provided in the manuscript.

Field-specific reporting

Please select the one below that is the best fit for your research. If you are not sure, read the appropriate sections before making your selection.

☒ Life sciences ☐ Behavioural & social sciences ☐ Ecological, evolutionary & environmental sciences

For a reference copy of the document with all sections, see nature.com/documents/nr-reporting-summary-flat.pdf

Life sciences study design

All studies must disclose on these points even when the disclosure is negative.

Sample size	No sample size was predetermined. Sample size was estimated by robustness of phenotype based on our preliminary experiments and previous work (Zheng et al. Science 2020 Nov 27;370(6520)). proper negative and whenever possible positive controls were used for each experiment.
Data exclusions	No data exclusions occurred in this study.
Replication	All key in vitro experiments were performed at least 3 times and all replication attempts were successful.
Randomization	cells were always randomly allocated into control and experimental groups. All samples used in each set of experiments were equal, except the experimental condition being tested.
Blinding	blinding was not done since this study relies on the investigator studying differences in cellines

Reporting for specific materials, systems and methods

We require information from authors about some types of materials, experimental systems and methods used in many studies. Here, indicate whether each material, system or method listed is relevant to your study. If you are not sure if a list item applies to your research, read the appropriate section before selecting a response.

Materials & experimental systems

n/a	Involved in the study
☐	☒ Antibodies
☐	☒ Eukaryotic cell lines
☒	☐ Palaeontology and archaeology
☒	☐ Animals and other organisms
☒	☐ Clinical data
☒	☐ Dual use research of concern

Methods

n/a	Involved in the study
☐	☒ ChIP-seq
☐	☒ Flow cytometry
☒	☐ MRI-based neuroimaging

Antibodies

Antibodies used

Anti-SSB1 (WB, 1:1000; IP, 2ug; ChIP, 2ug) Proteintech Cat. # 14809-1-AP
Anti-SSB2(WB, 1:1000) Proteintech Cat. # 16719-1-AP
Anti-INIP(WB, 1:300) sino biological Cat: # 204969-T10
Anti-INTS1(WB, 1:1000) Bethyl Laboratories Cat. # A300361A
Anti-INTS2(WB, 1:500) Santa Cruz Cat. # sc-514945
Anti-INTS3(WB, 1:2000; IP, 2ug; ChIP, 2ug) Proteintech Cat. # 16620-1-AP
Anti-INTS4(WB, 1:1000) Proteintech Cat. # 16130-1-AP
Anti-INTS5(WB, 1:2000; IP, 2ug; ChIP, 2ug) Proteintech Cat. # 14069-1-AP
Anti-INTS6(WB, 1:1000) Santa Cruz Cat. # sc-376524
Anti-INTS9(WB, 1:1000) Cell Signaling technology Cat. # 13945S
Anti-INTS10(WB, 1:1000) Proteintech Cat. # 15271-1-AP
Anti-INTS11(WB, 1:1000) Bethyl Laboratories Cat. # A301-274A
Anti-PP2A-A(WB, 1:1000) Proteintech Cat. # 15882-1-AP
Anti-PP2A-C(WB, 1:1000) Proteintech Cat. # 13482-1-AP
Anti-XRN2(WB, 1:1000; ChIP, 2ug) Proteintech Cat. # 11267-1-AP
Anti-DIS3(WB, 1:1000; ChIP, 2ug) Bethyl Laboratories Cat. # A303-765A
Anti-EXOSC10(WB, 1:1000; ChIP, 2ug) Proteintech Cat. # 16731-1-AP
Anti-MTR4(WB, 1:1000; ChIP, 2ug) Proteintech Cat. # 12719-2-AP
Anti-Tubulin(WB, 1:5000) Abclonal Cat. # AC008
V5 Tag Monoclonal Antibody (E10/V4RR)(WB, 1:5000; IP, 2ug) ThermoFisher Scientific Cat. # MA5-15253
Anti-H3K27ac(ChIP, 2ug) Abclonal Cat. # A7253
Anti-H3K4me1(ChIP, 2ug) Abclonal Cat. # A2355
Anti-H3K4me3(ChIP, 2ug) Abclonal Cat. # A2357
Anti-Rabbit IgG(IP, 2ug) Proteintech Cat. # B900610
Anti-Mouse IgG(IP, 2ug) Proteintech Cat. # B900620
Anti-Phospho-Rpb1CTD (Ser5) (D9N5I)(WB, 1:1000; ChIP, 2ug) Cell Signaling technology Cat. # 13523S
Anti-γH2AX(WB, 1:2000; CUT&Tag, 2ug; IF: 1:200) Bethyl Laboratories Cat. # A700-053
Anti-DYKDDDDK (Flag)(WB, 1:5000; IP, 2ug; IF, 1:100; ChIP, 2ug) Abclonal Cat. # AE005
Anti-S9.6(CUT&Tag, 2ug) Active Motif Cat. # 65683
Anti-Strep(WB, 1:1000) Abclonal Cat. # AE066
Anti-GST(WB, 1:1000) Huabio Cat. # EM80701
Alexa Fluor 488 conjugated goat anti-mouse IgG(IF, 1:1000) Yeasen Cat. # 33206ES60
Alexa Fluor 488 conjugated goat anti-rabbit IgG(IF, 1:1000) Yeasen Cat. # 33106ES60
Rhodamine (TRITC) Goat Anti-Mouse IgG (IF, 1:1000) Yeasen Cat. # 33209ES60
Rhodamine (TRITC) Goat Anti-Rabbit IgG(IF, 1:1000) Yeasen Cat. # 33109ES60
Mouse anti-rabbit IgG(CUT&Tag, 1:100) Solarbio Cat. # SPA231
Rabbit anti-mouse IgG(CUT&Tag, 1:100) Solarbio Cat. # K0034M
Anti-IdU(DNA fiber assay, 1:200) ThermoFisher Scientific Cat. # MA5-24879
Anti-BrdU(DNA fiber assay, 1:200) Abcam Cat. # AB6326

Validation

All these antibodies were commercially obtained and validated by vendors and multiple published studies, see manufacture's website for references. in addition, for key antibodies such as SSB1,SSB2 were validated by knock out or pooled knock out as negative control. other INTAC subunits antibodies were validated in our previous work (Zheng et al. Science 2020 Nov 27;370(6520)).

Eukaryotic cell lines

Policy information about cell lines and Sex and Gender in Research

Cell line source(s)

cell lines (SSB2 single KO, SSB2/1 double KO, SSB1-dTAG, INTS11-dTAG) were generated from DLD-1 cell line . DLD-1 cell was a gift from ALi shilatifard lab (Chikago, nortwestern university) who purchased it from ATCC. HEK Expi293 cell line were gifted from Yanhui Xu lab who purchased it from ATCC.

Authentication

Cells were cultured in media recommended by the vendors. We froze down stocks upon receiving the cell lines, and all experiments will be conducted on cells that have been passaged no more than 10 times. SSB2 single KO, SSB2/1 double KO were authenticated by western blot. SSB1-dTAG ,INTS11-dTAG cell line were authenticated by PCR and western blot. all rescue cell lines in SSB1-dTAG cell line were authenticated by western blot.

Mycoplasma contamination

All cell lines tested for Mycoplasma contamination every month.

Commonly misidentified lines
(See ICLAC register)

No commonly misidentified lines were used.

ChIP-seq

Data deposition

☒ Confirm that both raw and final processed data have been deposited in a public database such as GEO.

☒ Confirm that you have deposited or provided access to graph files (e.g. BED files) for the called peaks.

Data access links
May remain private before publication.

https://www.ncbi.nlm.nih.gov/geo/query/acc.cgi?acc=GSE223997

Files in database submission

GSM7009573 ChIP-H3K27ac_WT
GSM7009574 ChIP-H3K4me1_WT
GSM7009575 ChIP-Input_CTR-rep1
GSM7009576 ChIP-Input_CTR-rep2
GSM7009577 ChIP-Input_DKO-rep1
GSM7009578 ChIP-Input_DKO-rep2
GSM7009579 ChIP-Input_INTS2KO-rep1
GSM7009580 ChIP-Input_INTS2KO-rep2
GSM7009581 ChIP-Input_RNaseH1-DMSO
GSM7009582 ChIP-Input_RNaseH1-DOX
GSM7009583 ChIP-Input_sgCtr-rep1
GSM7009584 ChIP-Input_sgCtr-rep2
GSM7009585 ChIP-INTS3_CTR-rep1
GSM7009586 ChIP-INTS3_CTR-rep2
GSM7009587 ChIP-INTS3_DKO-rep1
GSM7009588 ChIP-INTS3_DKO-rep2
GSM7009589 ChIP-INTS3_RNaseH1-DMSO
GSM7009590 ChIP-INTS3_RNaseH1-DOX
GSM7009591 ChIP-INTS3_WT-rep1
GSM7009592 ChIP-INTS3_WT-rep2
GSM7009593 ChIP-INTS5_CTR-rep1
GSM7009594 ChIP-INTS5_CTR-rep2
GSM7009595 ChIP-INTS5_DKO-rep1
GSM7009596 ChIP-INTS5_DKO-rep2
GSM7009597 ChIP-INTS5_WT-rep1
GSM7009598 ChIP-INTS5_WT-rep2
GSM7009599 ChIP-polII-NTD_CTR-rep1
GSM7009600 ChIP-polII-NTD_DKO-rep1
GSM7009601 ChIP-polII-NTD_DKO-rep2
GSM7009602 ChIP-polII-NTD_INTS2KO-rep1
GSM7009603 ChIP-polII-NTD_INTS2KO-rep2
GSM7009604 ChIP-polII-NTD_sgCtr-rep1
GSM7009605 ChIP-polII-NTD_sgCtr-rep2
GSM7009606 ChIP-polII-pSer5_CTR-rep1
GSM7009607 ChIP-polII-pSer5_CTR-rep2
GSM7009608 ChIP-polII-pSer5_DKO-rep1
GSM7009609 ChIP-polII-pSer5_DKO-rep2
GSM7009610 ChIP-polII-pSer5_INTS2KO-rep1
GSM7009611 ChIP-polII-pSer5_INTS2KO-rep2
GSM7009612 ChIP-polII-pSer5_sgCtr-rep1
GSM7009613 ChIP-polII-pSer5_sgCtr-rep2
GSM7009614 ChIP-SSB1_CTR-rep1
GSM7009615 ChIP-SSB1_CTR-rep2
GSM7009616 ChIP-SSB1_DKO-rep1
GSM7009617 ChIP-SSB1_DKO-rep2
GSM7009618 ChIP-SSB1_RNaseH1-DMSO
GSM7009619 ChIP-SSB1_RNaseH1-DOX
GSM7009620 ChIP-SSB1_WT-rep1
GSM7009621 ChIP-SSB1_WT-rep2

Genome browser session
(e.g. UCSC)

no longer applicable

Methodology

Replicates

Two biological replicates for all next-generation sequencing assays. Most replication attempts were successful.

Sequencing depth

Experiment Total_reads Mapped_reads paired_or_single
ChIP-H3K27ac_WT 34386162 25400290 paired-end
ChIP-H3K4me1_WT 67740324 61197309 paired-end

ChIP-INTS3_WT-rep1 61111200 42982933 paired-end
ChIP-INTS3_WT-rep2 54636934 40997251 paired-end
ChIP-INTS5_WT-rep1 64929820 47559737 paired-end
ChIP-INTS5_WT-rep2 50327662 37935209 paired-end
ChIP-SSB1_WT-rep1 56914618 42028409 paired-end
ChIP-SSB1_WT-rep2 64059192 49107486 paired-end
ChIP-SSB1_RNaseH1-DOX 57227964 49531601 paired-end
ChIP-SSB1_RNaseH1-DMSO 49184466 42439246 paired-end
ChIP-INTS3_RNaseH1-DOX 57668212 48020248 paired-end
ChIP-INTS3_RNaseH1-DMSO 57507732 48865122 paired-end
ChIP-INTS3_DKO-rep1 83293772 64896365 paired-end
ChIP-INTS3_CTR-rep1 45954694 38904877 paired-end
ChIP-INTS5_DKO-rep1 70733478 57259021 paired-end
ChIP-INTS5_CTR-rep1 49765950 41948072 paired-end
ChIP-SSB1_DKO-rep1 71510928 55547819 paired-end
ChIP-SSB1_CTR-rep1 38565858 33180987 paired-end
ChIP-INTS3_DKO-rep2 49993702 38254882 paired-end
ChIP-INTS3_CTR-rep2 56256174 46334771 paired-end
ChIP-INTS5_CTR-rep2 97567818 86752833 paired-end
ChIP-INTS5_DKO-rep2 77418878 67633730 paired-end
ChIP-SSB1_DKO-rep2 33969230 29784057 paired-end
ChIP-SSB1_CTR-rep2 53014482 47735092 paired-end
ChIP-polII-NTD_CTR-rep1 50741734 42489741 paired-end
ChIP-polII-NTD_DKO-rep2 52704998 44642137 paired-end
ChIP-polII-NTD_CTR-rep1 57975194 48550095 paired-end
ChIP-polII-NTD_INTS2KO-rep1 94371462 86506303 paired-end
ChIP-polII-NTD_sgCtr-rep1 68007300 61152353 paired-end
ChIP-polII-NTD_INTS2KO-rep2 61953164 51956438 paired-end
ChIP-polII-NTD_sgCtr-rep2 52508578 44312944 paired-end
ChIP-polII-pSer5_INTS2KO-rep1 54104288 46678714 paired-end
ChIP-polII-pSer5_sgCtr-rep1 46304356 37169083 paired-end
ChIP-polII-pSer5_DKO-rep1 71650252 62088506 paired-end
ChIP-polII-pSer5_CTR-rep1 70624292 57028670 paired-end
ChIP-polII-pSer5_INTS2KO-rep2 79133512 70577984 paired-end
ChIP-polII-pSer5_CTR-rep2 54497894 41170575 paired-end
ChIP-polII-pSer5_DKO-rep2 40652272 28648739 paired-end
ChIP-polII-pSer5_sgCtr-rep2 91952552 78205003 paired-end

Antibodies

SSB1 (14809-1-AP, Proteintech), INTS3 (16620-1-AP, Proteintech), INTS5 (14069-1-AP, Proteintech), Pol II (NTD) (14958, Cell Signaling), Pol II (pSer5) (13523, Cell Signaling), H3K27ac (A7253, Abclonal).

Peak calling parameters

macs2 callpeak -f BAMPE -g hs --nomodel

Data quality

mapping rate > 70%, number of peaks > 20000

Software

ChIP-Rx analyses used Trim Galore v0.6.6, Bowtie v2.4.4, SAMtools v1.12, Picard Tools v2.25.5, MACS2 v2.2.7.1, R package ChIPseeker v1.28.3, deepTools v3.5.1, bedTools v2.30.0, R package BRGenomics v1.4.0

Flow Cytometry

Plots

Confirm that:

☒ The axis labels state the marker and fluorochrome used (e.g. CD4-FITC).

☒ The axis scales are clearly visible. Include numbers along axes only for bottom left plot of group (a 'group' is an analysis of identical markers).

☒ All plots are contour plots with outliers or pseudocolor plots.

☒ A numerical value for number of cells or percentage (with statistics) is provided.

Methodology

Sample preparation

Single-cell suspension of CTR and DKO were incubated with 70% ethanol in –20°C for 2 h. Following twice of PBS wash, cells were fixed with 4% formaldehyde (PFA) for 15 min. Next, cells were permeabilized with 0.25% Triton X-100 in PBS for 15 min and blocked with 2% BSA in PBS for 30 min. For intracellular γH2AX staining, 1 × 106 cells were incubated with 1 μg γH2AX antibody (Thermo) overnight at 4°C, followed by incubation with Alexa Fluor 488-conjugated secondary antibody for 30 min at room temperature. After washing with PBS for 3 times, cells were treated with PI staining buffer (Sangon Biotech) according to the manufacturer's protocol.

Instrument

CytoFLEX Flow Cytometer (Beckman Coulter).

Software

FACSDiva Flow Cytometry Software (BD Biosciences), FlowJo (TreeStar).

Cell population abundance	The initial cell population is greater than 30,000 and sorted cells with a purity >90% were subject to the following experiments. After sorting, sorted cells were re-run with the exact setting on the same instruments.
Gating strategy	Forward versus side scatter gating was used to identify cells and exclude debris and dead cells. A forward scatter height vs. forward scatter area density plot was used to exclude doublets. The PI (Propidium Iodide) signal was used to separate cells into G1, S, and G2/M phases.

☒ Tick this box to confirm that a figure exemplifying the gating strategy is provided in the Supplementary Information.
