# Supplementary material for: Author Correction: R-loop-dependent promoter-proximal termination ensures genome stability
Source: Nature. 2025 Jan 20;638(8049):E1. doi: 10.1038/s41586-025-08606-x (PMC11798841; doi:10.1038/s41586-025-08606-x)
Supplement: Supplementary file 1 — Original and corrected Figs. 1 and 2 and Supplementary Fig. 1 [file 41586_2025_8606_MOESM1_ESM.pdf]

nature portfolio

https://doi.org/10.1038/s41586-025-08606-x

Supplementary information

Author Correction: R-loop-dependent promoter-proximal termination ensures genome stability

In the format provided by the authors and unedited

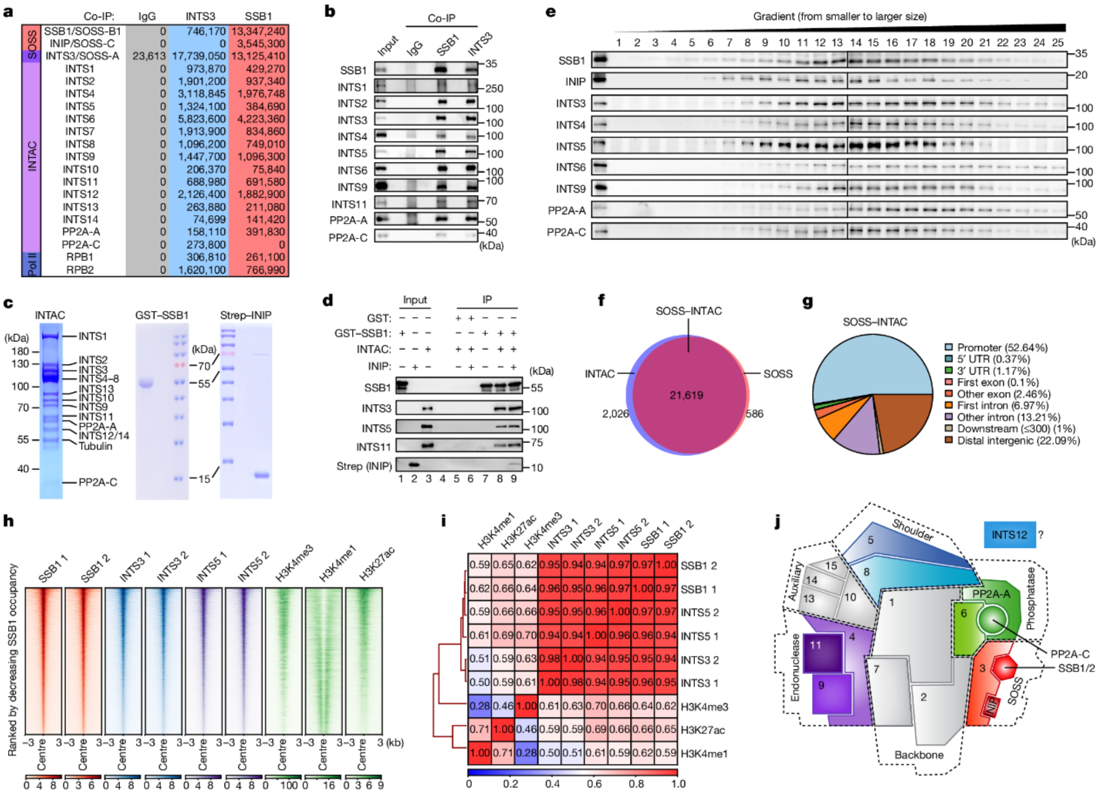

Original Fig. 1

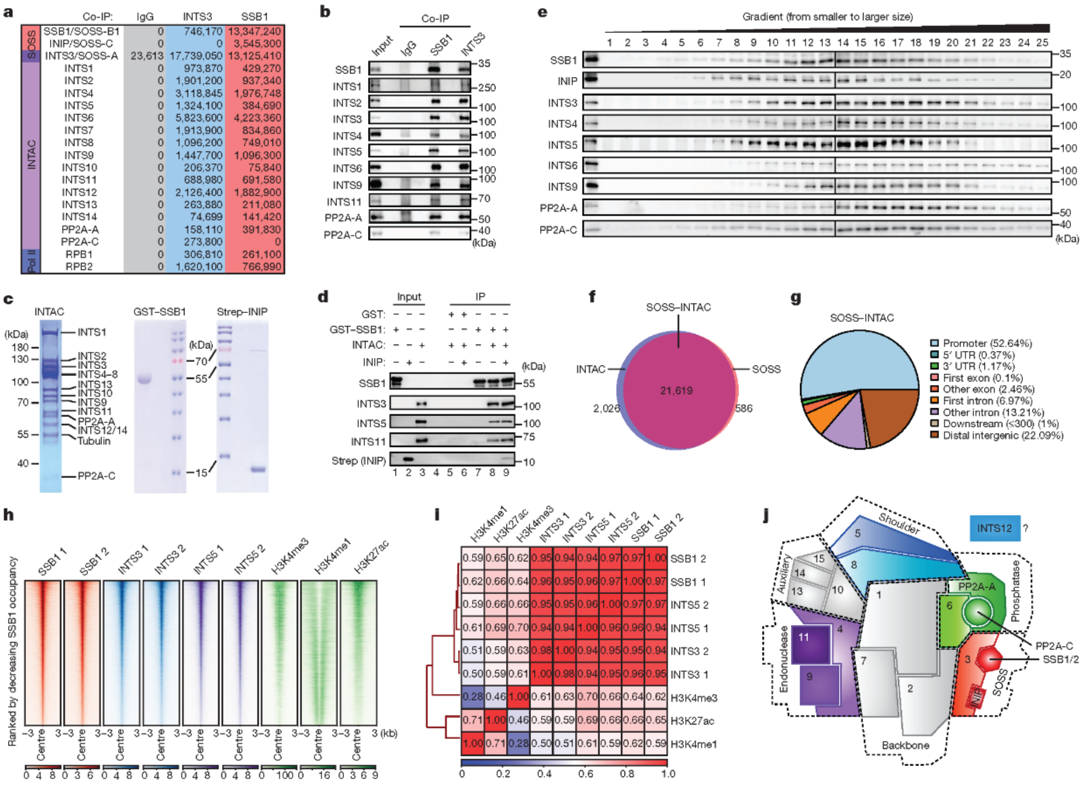

Corrected Fig. 1

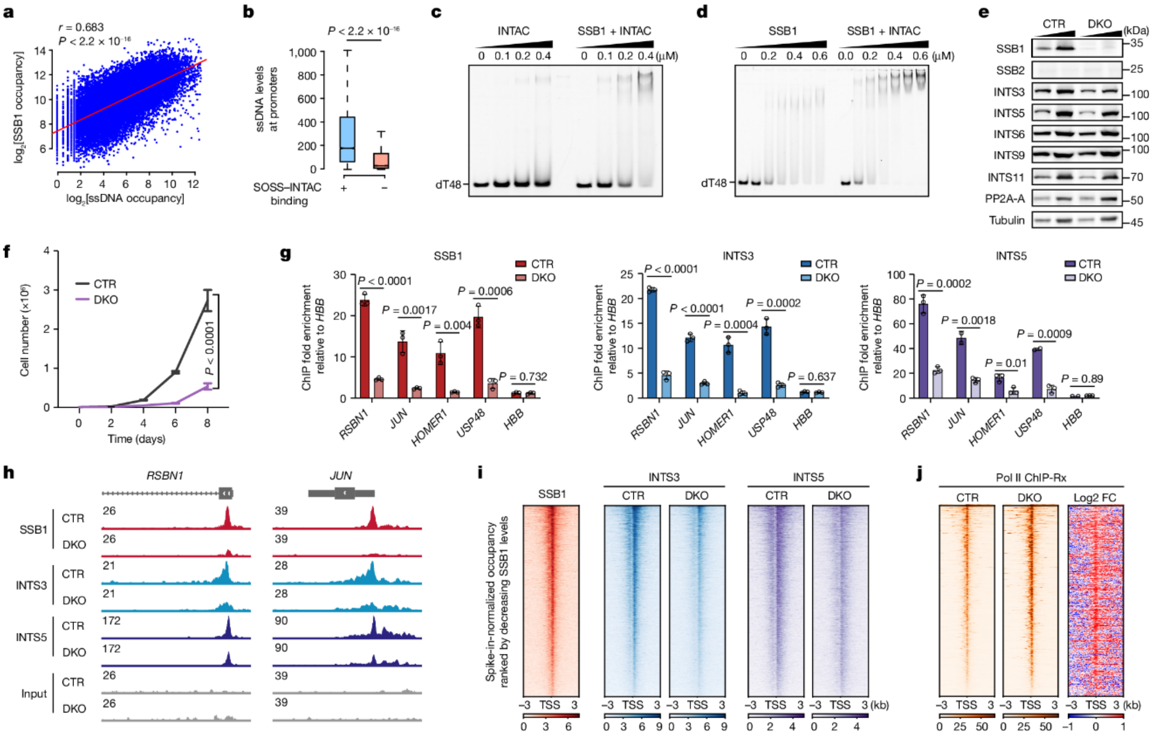

Original Fig. 2

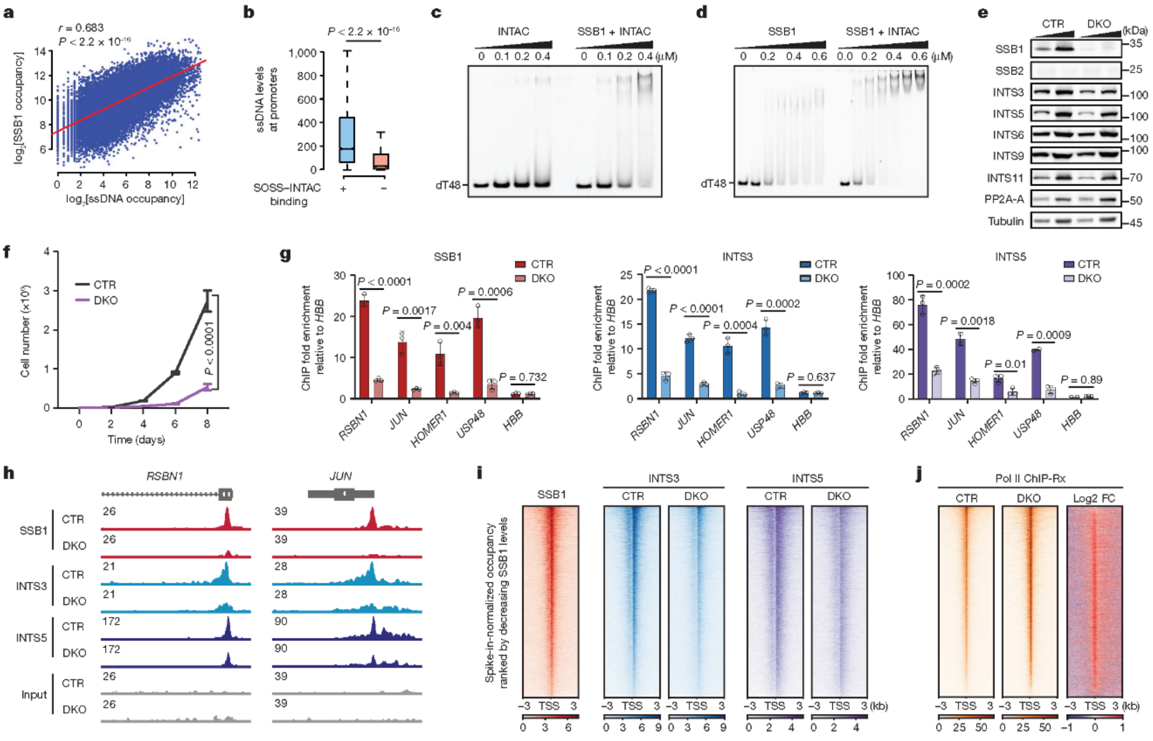

Corrected Fig. 2

Original Supplementary Figure 1

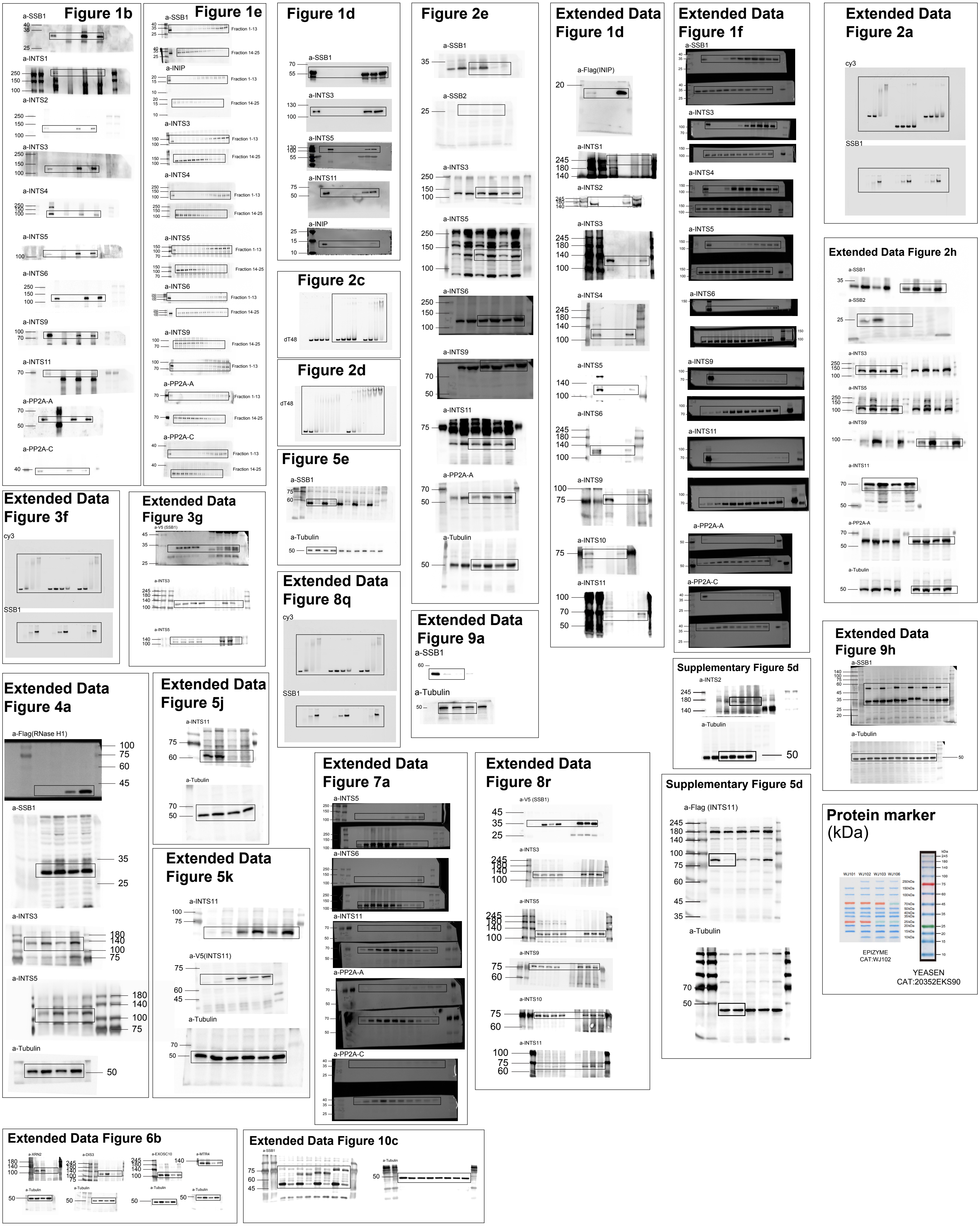

Supplementary Fig. 1. Source Data of unprocessed western blots.

Corrected Supplementary Figure 1

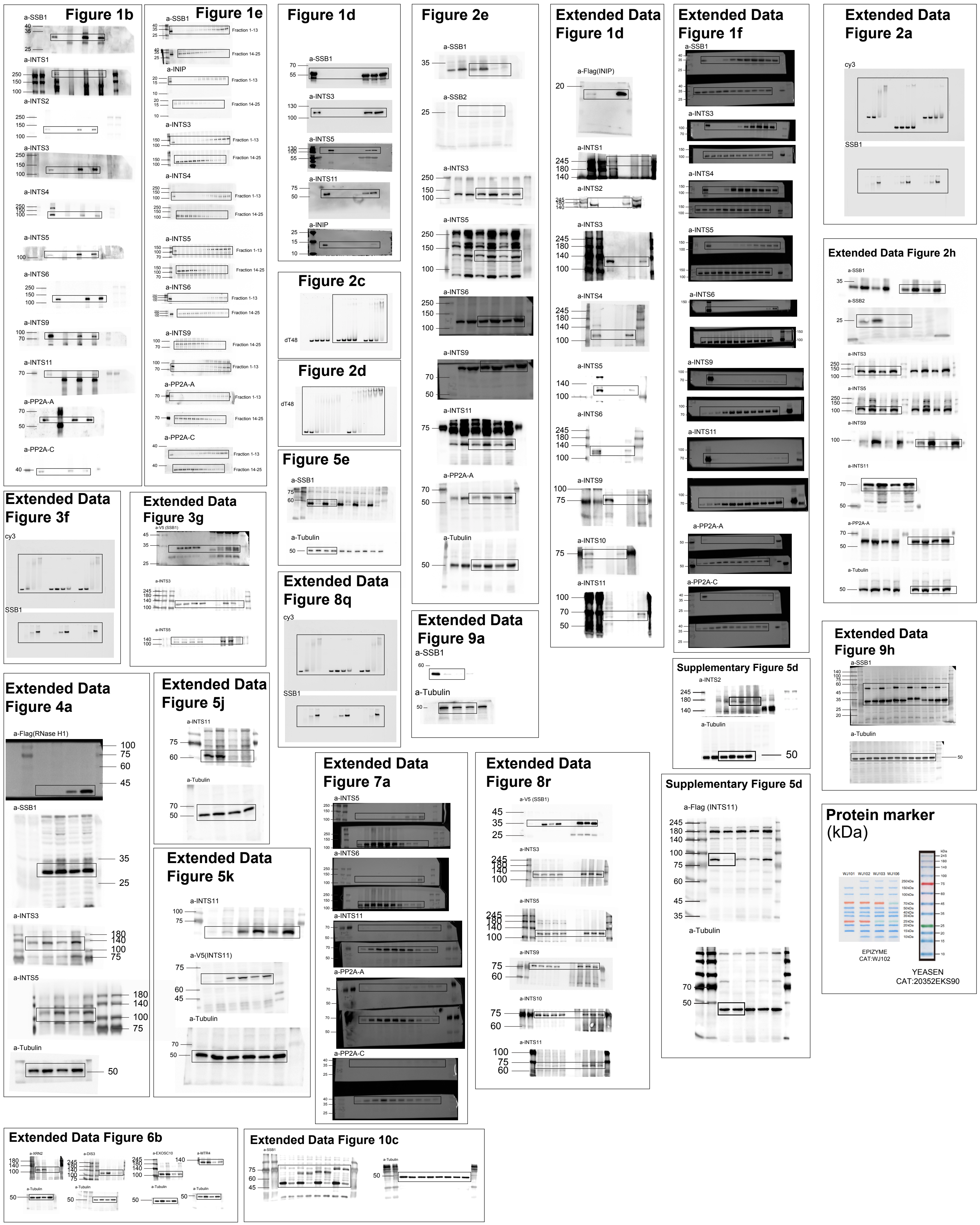

Supplementary Fig. 1. Source Data of unprocessed western blots.
